# Comparison of Minimally Invasive Colectomy Between Intra‐ Versus Extra‐Corporeal Anastomosis Technique (COMMIT Study): Mid‐Term Analysis

**DOI:** 10.1002/ags3.70184

**Published:** 2026-01-27

**Authors:** Takuya Takami, Takehito Yamamoto, Yoshiro Itatani, Ryosuke Mizuno, Shinya Hamasu, Keita Hanada, Kenji Kawada, Teppei Murakami, Satoshi Nagayama, Kazutaka Obama

**Affiliations:** ^1^ Department of Surgery, Graduate School of Medicine Kyoto University Kyoto Japan; ^2^ Department of Surgery Japanese Red Cross Otsu Hospital Otsu Shiga Japan; ^3^ Department of Surgery Rakuwakai Otowa Hospital Kyoto Japan; ^4^ Department of Surgery Kurashiki Central Hospital Okayama Japan; ^5^ Department of Surgery Kobe City Medical Center West Hospital Kobe Hyogo Japan; ^6^ Department of Surgery Uji‐Tokushukai Medical Center Kyoto Japan

**Keywords:** anastomosis, colon cancer, minimally invasive surgery, propensity score matching, recurrence‐free survival

## Abstract

**Aim:**

The adoption of intracorporeal anastomosis (IA) in minimally invasive colectomy for colon cancer has increased; however, its mid‐term oncological outcomes compared with those of extracorporeal anastomosis (EA) remain unclear.

**Methods:**

This multicenter retrospective cohort study analyzed patients who underwent minimally invasive curative colectomy for colon cancer between 2018 and 2022 from the Kyoto Colorectal Surgery Group database. Propensity score matching (1:4) was applied to balance patient, tumor, and surgical characteristics between IA and EA groups. Primary outcome was 2‐year recurrence‐free survival (RFS). Secondary outcomes included 2‐year cumulative incidence of peritoneal, anastomotic, and distant recurrence, and postoperative complications.

**Results:**

Among 1904 eligible patients (IA, *n* = 253; EA, *n* = 1651), matching yielded 224 IA and 862 EA cases with well‐balanced baseline covariates. After matching, the 2‐year RFS tended to be better in the IA group (hazard ratio [HR], 0.66; 95% confidence interval [CI], 0.41–1.06), though not statistically significant. The incidences of peritoneal and anastomotic recurrence were similar between the groups (risk difference [RD], 0.13%; 95% CI, −0.76% to 1.02%; and RD, −0.25%; 95% CI, −0.61% to 0.10%, respectively), whereas distant recurrence was lower in the IA group (RD, −4.79%; 95% CI, −6.75% to −2.83%). Subgroup analyses suggested a greater reduction in distant recurrence in patients with advanced tumors.

**Conclusion:**

IA demonstrated a comparable risk of peritoneal and anastomotic recurrence and suggested a potential reduction in distant recurrence without an associated deterioration in 2‐year RFS. These findings support IA as an oncologically reasonable option, even for advanced‐stage tumors.

## Graphical abstract

This multicenter retrospective cohort study compared mid‐term oncological outcomes between intracorporeal anastomosis (IA) and extracorporeal anastomosis (EA) in minimally invasive colectomy for colon cancer using propensity score–matched data from 2018 to 2022. After matching, IA showed a nonsignificant trend toward better 2‐year recurrence‐free survival and similar rates of peritoneal and anastomotic recurrence, while distant recurrence was lower in the IA group, particularly in patients with advanced tumors. Overall, IA demonstrated comparable oncological safety, supporting its use as a reasonable option even for advanced‐stage colon cancer.

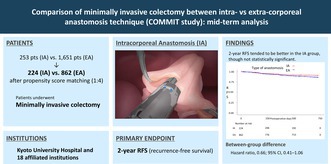

## 1 Introduction

Minimally invasive surgery is the global standard for the treatment of colon cancer [1]. In the early stages of its adoption, extracorporeal anastomosis (EA) was the predominant bowel reconstruction technique. However, since around 2010, there has been an increasing trend towards intracorporeal anastomosis (IA), where both colon resection and anastomosis are completed entirely within the abdominal cavity [2].

IA is generally considered more protective than EA because it reduces bowel traction and venous congestion [3]. However, IA requires precise manipulation and advanced surgical skills [4]. Furthermore, there are concerns regarding the potential for intraperitoneal dissemination of bacteria and tumor cells with IA.

Accumulating evidence exists regarding operative and postoperative short‐term outcomes of IA and EA. Namely, IA is associated with prolonged operative time, less operative bleeding, smaller incision, adequate resection margins, and higher number of harvested lymph nodes as operative outcomes [3, 5, 6, 7]. Furthermore, IA has been linked to milder postoperative pain, earlier bowel function recovery, and shorter length of hospital stay as short‐term postoperative outcomes [8, 9]. The incidence of postoperative complications, such as surgical site infection and anastomotic leakage, has been reported to be comparable between IA and EA [10, 11].

However, only a few studies have reported mid‐ and long‐term outcomes [12, 13, 14, 15, 16, 17, 18]. Although these studies suggest that the mid‐ and long‐term outcomes of IA are comparable with those of EA, most were single‐center investigations with limited sample sizes. Therefore, we compared the mid‐term outcomes of IA and EA using a multicenter database. We hypothesized that the mid‐term outcomes of IA would be noninferior to those of EA.

## 2 Materials and Methods

### 2.1 Study Design and Setting

This retrospective cohort study used the Kyoto Colorectal Surgery Group database, which contains data on patients with colorectal cancer who underwent surgery at the Kyoto University Hospital and 18 affiliated institutions since 2018. This database has already been used in some studies in the field of colorectal surgery [19, 20, 21, 22].

The study protocol was approved by the Ethics Committee of Kyoto University Graduate School and Faculty of Medicine (approval number: R0286). Informed consent was obtained using an opt‐out form available on the website. Patients who declined to participate were excluded.

### 2.2 Selection of Participants

We identified patients with colon cancer who underwent minimally invasive surgery (laparoscopic or robot‐assisted surgery) between January 2018 and December 2022. Inclusion criteria were as follows: (i) patients with cecal, ascending, transverse, descending, or sigmoid colon cancer who underwent curative resection with D2 or D3 lymph node dissection (LD); (ii) clinical stage 0–III; and (iii) availability of data on anastomotic approach (IA or EA). LD and clinical stage were defined according to the Japanese Classification of Colorectal, Appendiceal, and Anal Carcinoma [23]. Exclusion criteria were as follows: (i) emergency surgery; (ii) construction of a diverting stoma; or (iii) transanal double‐ or single‐stapling technique anastomosis.

### 2.3 Measurements

We selected the following baseline covariates: (i) patient‐related factors: age, sex, body mass index, American Society of Anesthesiologists Physical Status Classification System (ASA‐PS), diabetes mellitus, respiratory disease, hypertension, heart failure, preoperative carcinoembryonic antigen level, and neoadjuvant chemotherapy; (ii) tumor‐related factors: tumor location, tumor diameter, presence and type of preoperative decompression, clinical depth of tumor invasion (cT), and clinical lymph node metastasis (cN); and (iii) surgery‐related factors: surgical approach (laparoscopic or robot‐assisted surgery), surgical procedure, and extent of LD. Definitions of tumor location, cT, cN, surgical procedure, and extent of LD followed the Japanese Classification of Colorectal, Appendiceal, and Anal Carcinoma [23].

### 2.4 Outcomes

Primary outcome was 2‐year recurrence‐free survival (RFS). RFS was measured from surgery date to the date of cancer recurrence or death from any cause. Postoperative follow‐up was conducted in accordance with the Japanese Society for Cancer of the Colon and Rectum guidelines [24]. We selected 2‐year RFS because the majority of recurrences after curative colon surgery have been reported to occur within 2 years postoperatively [25]. Secondary outcomes included 2‐year cumulative incidences of peritoneal, anastomotic, and distant recurrences, and postoperative complications. Recurrence was classified based on the site of first recurrence. Although peritoneal and anastomotic recurrences are sometimes grouped together as local recurrence, they were analyzed separately in this study because the underlying mechanisms of recurrence are considered to be distinct. Distant recurrence was defined as the dissemination of tumor cells via the bloodstream, leading to metastases in organs, such as the liver or lungs, as well as lymph node metastasis. Postoperative complications were defined as events classified as Clavien–Dindo grade ≥ III within one postoperative month [26].

### 2.5 Statistical Analysis

We conducted a complete case analysis, excluding observations with missing values. To balance baseline characteristics between IA and EA groups, propensity scores for IA were estimated using a logistic regression model. We then performed 1:4 matching without replacement using a caliper of < 0.2 of the standardized difference in the logit of the propensity score. Variables included in the propensity score model comprised all patient‐, tumor‐, and surgery‐related factors, as described above. The baseline characteristics were compared before and after the matching using standardized mean differences (SMDs), with an SMD of ≤ 0.1 considered to indicate adequate covariate balance [27].

The primary outcome (2‐year RFS) was analyzed using survival analysis, with surgery date being the index date. Patients who were lost to follow‐up within 2 years postoperatively were censored. The Kaplan–Meier method was used for descriptive analysis, and hazard ratios (HRs) with 95% confidence intervals (CIs) were estimated using Cox proportional hazards regression. HRs were calculated using the EA group as the reference. The secondary outcomes, including 2‐year cumulative incidence of peritoneal recurrence, 2‐year cumulative incidence of anastomotic recurrence, 2‐year cumulative incidence of distant recurrence, and postoperative complications, were assessed by calculating risk differences (RDs) with corresponding 95% CIs. RDs were also calculated using the EA group as the reference. For the 2‐year cumulative incidence of peritoneal, anastomotic, and distant recurrence, RDs and their 95% CIs were calculated based on the cumulative incidence functions estimated using the Fine‐Gray model, in which patients lost to follow‐up within 2 years were censored and deaths within 2 years were treated as competing risks. This model estimates cumulative incidence by modeling the subdistribution hazard for recurrence without censoring patients who experience a competing risk of death [28]. We used absolute RDs instead of risk ratios because the incidences of peritoneal recurrence, anastomotic recurrence, distant recurrence, and postoperative complications were expected to be low.

Subgroup analyses were conducted according to tumor diameter (≤ 40 mm vs. > 40 mm), clinical depth of tumor invasion (cT3 or shallower vs. cT4), clinical lymph node metastasis status (cN− vs. cN+), and surgical procedure (right vs. left colectomy). The cutoff value of 40 mm for tumor diameter was chosen because it corresponded to the median tumor size in our cohort. All statistical analyses were performed using R version 4.4.1 (R Foundation for Statistical Computing, Vienna, Austria).

## 3 Results

### 3.1 Characteristics of Study Participants

**FIGURE 1:**
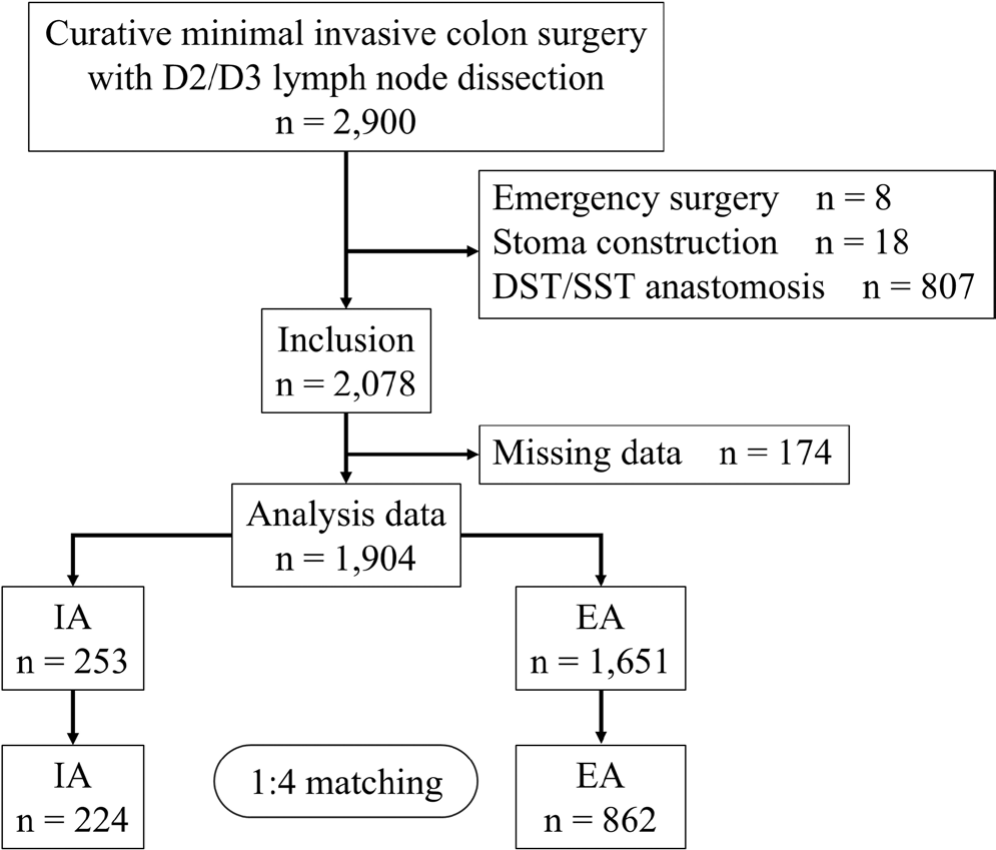
The participant flow chart of the study. DST, double stapling technique; EA, extracorporeal anastomosis; IA, intracorporeal anastomosis; SST, single stapling technique.

A total of 2900 patients underwent curative minimally invasive surgery for cecal, ascending, transverse, descending, or sigmoid colon cancer with D2 or D3 LD. Patients who underwent emergency surgery (*n* = 8), diverting stoma construction (*n* = 18), or double‐ or single‐stapling technique anastomosis (*n* = 807) were excluded. An additional 174 patients with missing data were excluded. Consequently, 1904 patients were included in the final analysis: 253 and 1651 in the IA and EA groups, respectively. Median follow‐up period was 32 months (interquartile range: 25–39 months) in the IA group and 36 months (interquartile range: 28–46 months) in the EA group. After a 1:4 matching, 224 and 862 patients were included in the IA and EA groups, respectively (Figure 1).

Table 1 shows the baseline characteristics of the two groups before and after matching. After matching, all baseline covariates were well balanced, as indicated by SMDs of ≤ 0.1. In addition, all cases performed with robotic surgery were excluded from the matched analysis. Postoperative pathological findings (pT and pN) are presented in Table 2 before and after matching for descriptive purposes, with SMDs shown for reference.

**TABLE 1:** Baseline characteristics before and after matching.

	Before matching			After matching		
	IA (*n* = 253)	EA (*n* = 1651)	SMD	IA (*n* = 224)	EA (*n* = 862)	SMD
Age (years), median [IQR]	74 [66–80]	75 [69–81]	0.151	74 [67–80]	74 [68–80]	0.008
Sex, *n* (%)						
Male	138 (54.5)	796 (48.2)	0.127	121 (54.0)	459 (53.2)	0.015
Female	115 (45.5)	855 (51.8)		103 (46.0)	403 (46.8)	
BMI (kg/m^2^), *n* (%)						
< 18.5	26 (10.3)	208 (12.6)	0.159	24 (10.7)	82 (9.5)	0.040
18.5–25	154 (60.9)	1077 (65.2)		139 (62.1)	540 (62.6)	
≥ 25	73 (28.9)	366 (22.2)		61 (27.2)	240 (27.8)	
ASA‐PS, *n* (%)						
1	43 (17.0)	302 (18.3)	0.098	39 (17.4)	155 (18.0)	0.045
2	175 (69.2)	1073 (65.0)		152 (67.9)	568 (65.9)	
3	33 (13.0)	264 (16.0)		31 (13.8)	130 (15.1)	
4	2 (0.8)	12 (0.7)		2 (0.9)	9 (1.0)	
Diabetes mellitus, *n* (%)	65 (25.7)	358 (21.7)	0.094	55 (24.6)	213 (24.7)	0.004
Respiratory disease, *n* (%)	13 (5.1)	81 (4.9)	0.011	13 (5.8)	49 (5.7)	0.005
Hypertension, *n* (%)	128 (50.6)	776 (47.0)	0.072	111 (49.6)	422 (49.0)	0.012
Heart failure, *n* (%)	3 (1.2)	14 (0.8)	0.034	3 (1.3)	11 (1.3)	0.006
CEA, *n* (%)						
≤ 5.0 ng/mL	175 (69.2)	1124 (68.1)	0.059	154 (68.8)	589 (68.3)	0.021
> 5.0 ng/mL	72 (28.5)	499 (30.2)		65 (29.0)	251 (29.1)	
Not tested	6 (2.4)	28 (1.7)		5 (2.2)	22 (2.6)	
Neoadjuvant chemotherapy, *n* (%)	4 (1.6)	6 (0.4)	0.124	1 (0.4)	6 (0.7)	0.033
Location, *n* (%)						
Cecum	35 (13.8)	312 (18.9)	0.214	33 (14.7)	129 (15.0)	0.086
Ascending	97 (38.3)	657 (39.8)		86 (38.4)	360 (41.8)	
Transverse	57 (22.5)	386 (23.4)		46 (20.5)	172 (20.0)	
Descending	41 (16.2)	170 (10.3)		37 (16.5)	121 (14.0)	
Sigmoid	23 (7.6)	126 (9.1)		22 (9.8)	80 (9.3)	
Tumor diameter (mm), median [IQR]	40 [25–52]	40 [25–56]	0.109	40 [25–50]	40 [24.25–55]	0.032
Preoperative decompression, *n* (%)						
Not performed	238 (94.1)	1506 (91.2)	0.129	210 (93.8)	813 (94.3)	0.065
Nasogastric decompression tube	0 (0.0)	1 (0.1)		0 (0.0)	0 (0.0)	
Nasointestinal decompression tube	1 (0.4)	10 (0.6)		1 (0.4)	6 (0.7)	
Transanal decompression tube	1 (0.4)	8 (0.5)		1 (0.4)	3 (0.3)	
Stent	8 (3.2)	92 (5.6)		7 (3.1)	27 (3.1)	
Others	5 (2.0)	34 (2.1)		5 (2.2)	13 (1.5)	
cT, *n* (%)						
Tis	1 (0.4)	9 (0.5)	0.159	1 (0.4)	4 (0.5)	0.028
T1	48 (19.0)	235 (14.2)		44 (29.6)	165 (19.1)	
T2	54 (21.3)	313 (19.0)		46 (20.5)	170 (19.7)	
T3	91 (36.0)	678 (41.1)		82 (36.6)	321 (37.2)	
T4	59 (25.2)	416 (23.3)		51 (22.8)	202 (23.4)	
cN, *n* (%)						
N0	142 (56.1)	975 (59.1)	0.150	129 (57.6)	496 (57.5)	0.023
N1	90 (35.6)	488 (29.6)		77 (34.4)	292 (33.9)	
N2	19 (7.5)	168 (10.2)		16 (7.1)	65 (7.5)	
N3	2 (0.8)	20 (1.2)		2 (0.9)	9 (1.0)	
Surgical approach, *n* (%)						
Laparoscopic	228 (90.1)	1649 (99.9)	0.459	224 (100.0)	862 (100.0)	< 0.001
Robot‐assisted	25 (9.9)	2 (0.1)		0 (0.0)	0 (0.0)	
Surgical procedure, *n* (%)						
Partial colectomy	29 (11.5)	175 (10.6)	0.148	22 (9.8)	91 (10.6)	0.083
Ileocecal colectomy	64 (25.3)	427 (25.9)		58 (25.9)	237 (27.5)	
Right hemicolectomy	99 (39.1)	739 (44.8)		88 (39.3)	348 (40.4)	
Left hemicolectomy	37 (14.6)	192 (11.6)		33 (14.7)	110 (12.8)	
Sigmoid colectomy	24 (9.5)	118 (7.1)		23 (10.3)	76 (8.8)	
Lymph node dissection, *n* (%)						
D2	21 (8.3)	211 (12.8)	0.146	21 (9.4)	81 (9.4)	0.001
D3	232 (91.7)	1440 (87.2)		203 (90.6)	781 (90.6)	

Abbreviations: ASA‐PS, American Society of Anesthesiologists Physical Status Classification System; BMI, body mass index; CEA, carcinoembryonic antigen; EA, extracorporeal anastomosis; IA, intracorporeal anastomosis; IQR, interquartile range; SMD, standardized mean difference.

**TABLE 2:** Pathological findings before and after matching.

	Before matching			After matching		
	IA (*n* = 253)	EA (*n* = 1651)	SMD	IA (*n* = 224)	EA (*n* = 862)	SMD
pT, *n* (%)						
Tis	10 (4.0)	37 (2.2)	0.230	8 (3.6)	20 (2.3)	0.119
T1	51 (20.2)	252 (15.3)		44 (19.6)	167 (19.4)	
T2	42 (16.6)	216 (13.1)		37 (16.5)	125 (14.5)	
T3	124 (49.0)	907 (54.9)		112 (50.0)	439 (50.9)	
T4	26 (10.3)	239 (14.5)		23 (10.3)	111 (12.9)	
pN, *n* (%)						
N0	190 (75.1)	1110 (67.2)	0.150	168 (75.0)	591 (68.6)	0.161
N1	50 (19.8)	381 (23.1)		44 (19.6)	199 (23.1)	
N2	11 (4.3)	128 (7.8)		10 (4.5)	56 (6.5)	
N3	2 (0.8)	32 (1.9)		2 (0.9)	16 (1.9)	

Abbreviations: EA, extracorporeal anastomosis; IA, intracorporeal anastomosis; SMD, standardized mean difference.

### 3.2 Primary Outcome

**FIGURE 2:**
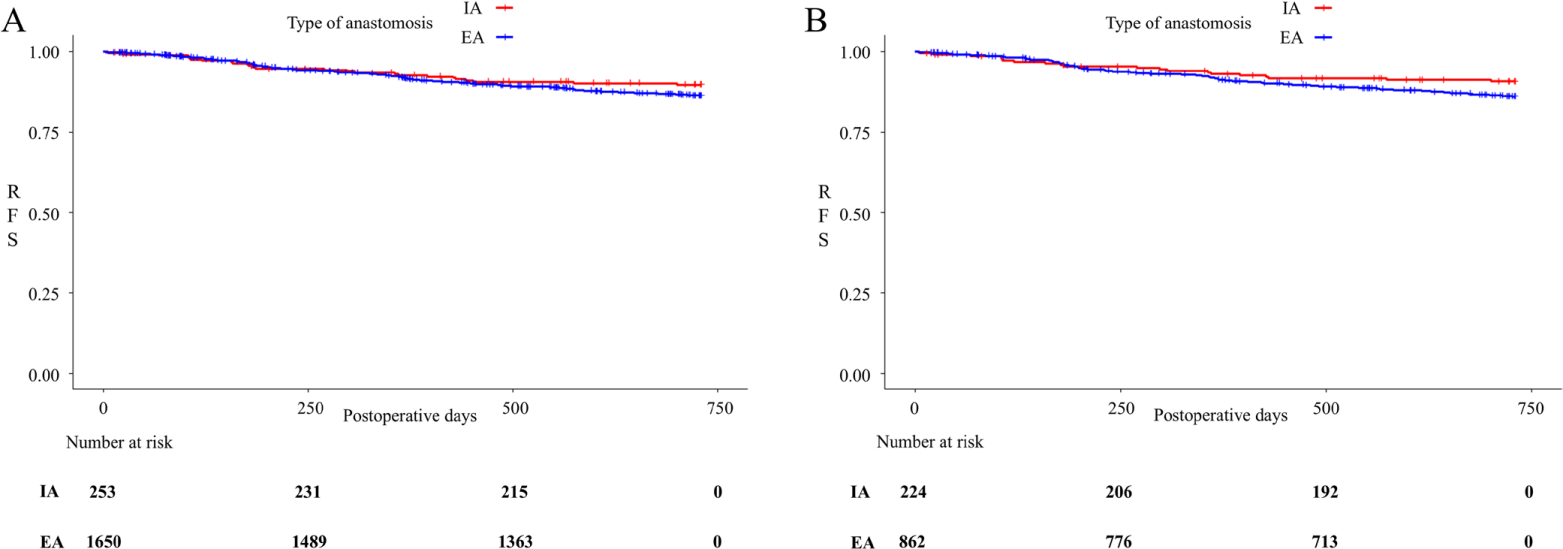
The Kaplan–Meier survival curves before and after matching. (A) Before matching, (B) After matching. EA, extracorporeal anastomosis; IA, intracorporeal anastomosis; RFS, recurrence‐free survival.

Before propensity score matching, the 2‐year RFS rates were 89.8% in the IA group and 86.4% in the EA group. After matching, the 2‐year RFS rates were 90.7% for IA and 86.0% for EA. Figure 2 displays the Kaplan–Meier survival curves for the 2‐year RFS before and after matching. As the 2‐year mark approached in both analyses, the RFS rate in the IA group slightly exceeded that in the EA group. The HR before matching was 0.75 (95% CI, 0.50–1.14); whereas after matching, it was 0.66 (95% CI, 0.41–1.06).

### 3.3 Secondary Outcomes

After matching, the 2‐year cumulative incidences of peritoneal and anastomotic recurrence did not differ significantly between the groups (RD, 0.13%; 95% CI, −0.76% to 1.02%; and RD, −0.25%; 95% CI, −0.61% to 0.10%). In contrast, the 2‐year cumulative incidence of distant recurrence in the IA group was lower than that in the EA group (RD, −4.79%; 95% CI, −6.75% to −2.83%) (Table 3). The incidence of postoperative complications was comparable between the two groups (Table 4).

**TABLE 3:** The 2‐year cumulative incidence of peritoneal, anastomotic, and distant recurrence before and after matching.

Outcome	Before matching	After matching
Peritoneal recurrence		
IA (%)	6/253 (2.4)	4/224 (1.8)
EA (%)	27/1651 (1.6)	14/862 (1.6)
RD (%) [95% CI]^a^	0.72 [0.07 to 1.36]	0.13 [−0.76 to 1.02]
Anastomotic recurrence		
IA (%)	0/253 (0.0)	0/224 (0.0)
EA (%)	8/1651 (0.5)	2/862 (0.2)
RD (%) [95% CI]^a^	−0.51 [−0.87 to −0.16]	−0.25 [−0.61 to 0.10]
Distant recurrence		
IA (%)	12/253 (4.7)	9/224 (4.0)
EA (%)	126/1651 (7.6)	73/862 (8.5)
RD (%) [95% CI]^a^	−3.18 [−4.53 to −1.82]	−4.79 [−6.75 to −2.83]

Abbreviations: CI, confidence interval; EA, extracorporeal anastomosis; IA, intracorporeal anastomosis; RD, risk difference.

^a^ RDs and 95% CIs were calculated from the cumulative incidence functions estimated by the Fine‐Gray model. RDs were calculated with EA as the reference group (IA–EA).

**TABLE 4:** Postoperative complications before and after matching.

Outcome	Before matching	After matching
SSI		
IA (%)	1/253 (0.4)	1/224 (0.4)
EA (%)	21/1651 (1.3)	13/862 (1.5)
RD (%) [95% CI]	−0.88 [−1.62 to 0.98]	−1.06 [−2.18 to 1.07]
Anastomotic leakage		
IA (%)	2/253 (0.8)	2/224 (0.9)
EA (%)	12/1651 (0.7)	8/862 (0.9)
RD (%) [95% CI]	−0.06 [−0.72 to 2.13]	−0.04 [−1.14 to 2.31]
Others		
IA (%)	2/253 (0.7)	2/224 (0.9)
EA (%)	11/1651 (0.8)	7/862 (0.8)
RD (%) [95% CI]	0.12 [−0.65 to 2.19]	0.08 [−0.99 to 2.42]
All		
IA (%)	4/253 (1.6)	4/224 (1.8)
EA (%)	30/1651 (1.8)	20/862 (2.3)
RD (%) [95% CI]	−0.24 [−1.47 to 2.24]	−0.53 [−2.18 to 2.30]

*Note:* RD were calculated with EA as the reference group (IA–EA).

Abbreviations: CI, confidence interval; EA, extracorporeal anastomosis; IA, intracorporeal anastomosis; RD, risk difference; SSI, surgical site infection.

### 3.4 Subgroup Analysis

**FIGURE 3:**
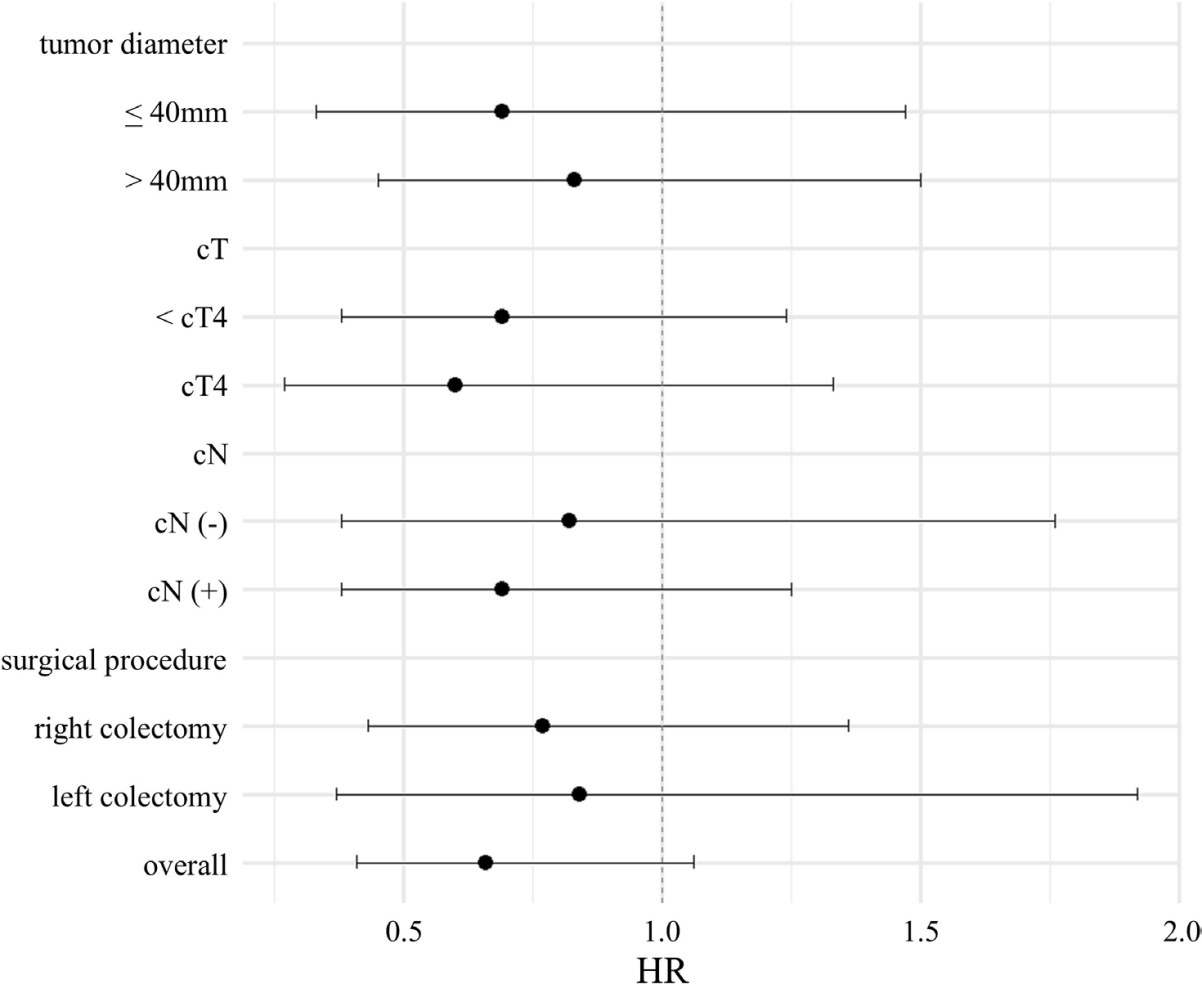
Subgroup analysis for RFS. HR were calculated with EA as the reference group (IA/EA). HR, hazard ratio; RFS, recurrence‐free survival.

**FIGURE 4:**
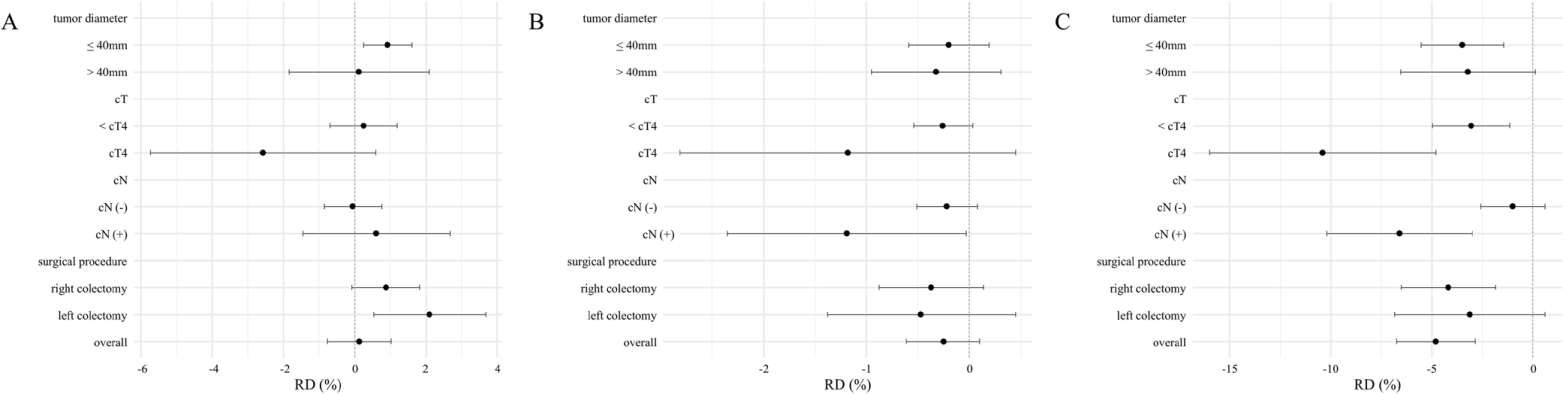
Subgroup analysis for peritoneal, anastomotic, and distant recurrence. (A) Peritoneal recurrence, (B) Anastomotic recurrence, (C) Distant recurrence. RD were calculated with EA as the reference group (IA–EA). RD, risk difference.

In the subgroup analyses, the RFS results were consistent across all subgroups (Figure 3). Across almost all subgroups, there were no statistically significant differences in the 2‐year cumulative incidences of peritoneal and anastomotic recurrence (Figure 4A,B). For the 2‐year cumulative distant recurrence, all the point estimates of RD were < 0%, with a more pronounced effect observed in patients with clinically advanced tumors (Figure 4C).

## 4 Discussion

To the best of our knowledge, this study included the largest number of institutions and participants to date among studies evaluating the mid‐ to long‐term outcomes of IA. In this cohort, the 2‐year cumulative incidence of peritoneal and anastomotic recurrence did not differ between the IA and EA groups, whereas the 2‐year cumulative incidence of distant recurrence was lower in the IA group. Consequently, the 2‐year RFS also tended to be better in the IA group, although the difference was not statistically significant. Notably, the 2‐year RFS observed in the IA group in the present study was comparable to that reported in previous studies [14, 15, 17, 18], which have generally shown 2‐year RFS rates of approximately 80%–90%, supporting the external validity of our findings.

Regarding peritoneal recurrence, a previous study reported that intraoperative peritoneal lavage cytology performed after completion of IA yielded no positive results [29]. Consistent with this, both the present study and previous reports found no difference in the incidence of peritoneal metastases between the IA and EA groups [14, 15, 17, 18]. Taken together, the concordance between the present findings and prior studies strengthens the validity of this result. In addition, a multicenter randomized controlled trial evaluating long‐term outcomes of IA versus EA is currently ongoing [30], and more robust evidence is anticipated.

No previous studies have described a reduction in the risk of distant recurrence in the IA group. In the present study, the IA group had a lower risk of distant recurrence. One possible factor to be considered is a slight tendency for pT and pN to be advanced disease in the EA group. However, the balances were acceptable (SMD < 0.2) [31], and their influence on the observed results is minimal. Furthermore, the choice between IA and EA in clinical practice is typically determined at the discretion of the surgeon, taking into account preoperative clinical findings. Therefore, the present results, which were obtained under conditions in which clinical tumor invasiveness and lymph node status were well balanced between the groups, retain meaningful interpretability.

In this study, postoperative complications, such as surgical site infection and anastomotic leakage, were comparable between the IA and EA groups, and these findings are consistent with those of previous reports [10, 11]. Therefore, IA can be considered safe when performed using the appropriate techniques. However, because this was a retrospective study, caution is warranted regarding the possibility of selection bias arising from an imbalance in patient distribution, as high‐risk patients, such as those with more severe preoperative bowel obstruction, may have been more frequently included in the EA group, although the presence and type of preoperative decompression were well balanced between the two groups after matching.

This study has some limitations. First, it may have been subject to bias from unmeasured confounders such as preoperative bowel preparation (mechanical preparation with or without chemical preparation) and surgeon‐related factors. Because of the retrospective nature of this cohort study, there were no predefined criteria for selecting between IA and EA, and the decision was left to the discretion of the operating surgeon in routine practice. In the IA group, procedures may have been more frequently performed by highly experienced surgeons, which could have influenced the outcomes. Although these factors can influence short‐term outcomes, their impact on mid‐term outcomes is likely to be limited. Second, the analysis was conducted using only cases without missing values, which may have introduced a selection bias. Third, this study evaluated outcomes over a 2‐year period; therefore, long‐term outcomes, such as 5‐year RFS, could not be assessed. Although longer follow‐up, for example 3 years or more, might provide additional information, extending the endpoint would have substantially reduced the number of patients with adequate follow‐up, resulting in a loss of statistical power and precision. Accordingly, 2‐year RFS was selected as the primary endpoint, as it represents a clinically relevant outcome while maintaining sufficient sample size and methodological robustness. Finally, the study population was restricted to cases from facilities included in the database, which may have limited the generalizability and transportability of the findings.

In conclusion, this study demonstrated that IA was not associated with worse 2‐year RFS compared with EA, indicating that IA does not compromise oncological outcomes. These findings suggest that IA represents an oncologically safe and reasonable surgical option, particularly in patients with advanced disease. Larger prospective randomized controlled trials are required to confirm these findings.

## Author Contributions

**Takuya Takami:** conceptualization, methodology, writing – review and editing, writing – original draft, data curation, software, formal analysis, validation, investigation. **Takehito Yamamoto:** conceptualization, methodology, writing – original draft, writing – review and editing, data curation, software, formal analysis, validation, investigation. **Yoshiro Itatani:** conceptualization, methodology, supervision, writing – review and editing, writing – original draft, project administration, funding acquisition. **Ryosuke Mizuno:** writing – review and editing, resources. **Shinya Hamasu:** writing – review and editing, resources. **Keita Hanada:** writing – review and editing, resources. **Kenji Kawada:** writing – review and editing, resources. **Teppei Murakami:** writing – review and editing, resources. **Satoshi Nagayama:** writing – review and editing, resources. **Kazutaka Obama:** supervision, writing – review and editing, project administration.

## Funding

The authors have nothing to report.

## Ethics Statement

The protocol for this research project has been approved by a suitably constituted Ethics Committee of the institution and it conforms to the provisions of the Declaration of Helsinki. Ethics Committee of Kyoto University Graduate School and Faculty of Medicine, Approval No. R0286.

## Consent

Informed consent was obtained via an opt‐out form on the website. Patients who declined to participate were excluded.

## Conflicts of Interest

The authors declare no conflicts of interest.
